# Joint deep autoencoder and subgraph augmentation for inferring microbial responses to drugs

**DOI:** 10.1093/bib/bbad483

**Published:** 2024-01-03

**Authors:** Zhecheng Zhou, Linlin Zhuo, Xiangzheng Fu, Quan Zou

**Affiliations:** School of Data Science and Artificial Intelligence, Wenzhou University of Technology, 325000, Wenzhou, China; School of Data Science and Artificial Intelligence, Wenzhou University of Technology, 325000, Wenzhou, China; College of Computer Science and Electronic Engineering, Hunan University, 410012, Changsha, China; Institute of Fundamental and Frontier Sciences, University of Electronic Science and Technology of China, 611730, Chengdu, China

**Keywords:** microbial responses to drugs, deep autoencoder, microbe and drug subgraphs, multi-hop neighborhood information

## Abstract

Exploring microbial stress responses to drugs is crucial for the advancement of new therapeutic methods. While current artificial intelligence methodologies have expedited our understanding of potential microbial responses to drugs, the models are constrained by the imprecise representation of microbes and drugs. To this end, we combine deep autoencoder and subgraph augmentation technology for the first time to propose a model called JDASA-MRD, which can identify the potential indistinguishable responses of microbes to drugs. In the JDASA-MRD model, we begin by feeding the established similarity matrices of microbe and drug into the deep autoencoder, enabling to extract robust initial features of both microbes and drugs. Subsequently, we employ the MinHash and HyperLogLog algorithms to account intersections and cardinality data between microbe and drug subgraphs, thus deeply extracting the multi-hop neighborhood information of nodes. Finally, by integrating the initial node features with subgraph topological information, we leverage graph neural network technology to predict the microbes’ responses to drugs, offering a more effective solution to the ’over-smoothing’ challenge. Comparative analyses on multiple public datasets confirm that the JDASA-MRD model’s performance surpasses that of current state-of-the-art models. This research aims to offer a more profound insight into the adaptability of microbes to drugs and to furnish pivotal guidance for drug treatment strategies. Our data and code are publicly available at: https://github.com/ZZCrazy00/JDASA-MRD.

## INTRODUCTION

Microbes encompass entities such as bacteria, fungi and viruses-minute organisms that reside both within and outside our bodies. Their response to drugs is multifaceted and intricate [1–3]. Beyond their roles in drug metabolism and detoxification [4], affecting both the efficacy and toxicity of medications [5], certain microbes are even capable of producing specialized enzymes [6]. These enzymes can transform drugs into active metabolites or inhibit their effectiveness [7], further influencing therapeutic outcomes [8–10]. Many ailments, be they infectious or chronic, can be linked to microbial infections [11]. To counteract these diseases, drugs such as antibiotics target and regulate microbial infections, with antibiotics specifically addressing bacterial infections [12]. An intricate understanding of how microbes react to drugs can significantly influence the choice and potency of treatments [13]. This intricate response of microbes to drugs weaves a vast network [14], encapsulating direct responses, like the inhibition of bacteria and fungi, the toxicity of drugs under microbial stress and indirect responses where drugs influence microbes via host immune responses or metabolic pathways [15]. Thus, the precise prediction of microbial responses to drugs holds immense promise for areas such as drug resistance studies, pharmaceutical advancement and the realm of personalized medicine [16, 17].

Conventional methodologies for probing microbial responses to drugs encompass laboratory experiments, clinical observations and empirical therapies [18, 19]. Laboratory investigations chiefly assess the vulnerability and resistance of microbes to drugs. This is done by culturing them *in vitro*, subjecting them to varying drug concentrations and then evaluating either the drugs’ inhibitory effects on microbial growth or the survival rates of the microbes [20, 21]. In the clinical realm, both observations and empirical treatments offer insights into the susceptibility or resilience of particular microbes to specific drugs [22]. On the computational front, statistics undertakes the task of analyzing microbial genomic data alongside drug chemical structures. Drawing upon extant data on microbial drug responses, it reveals potential responses of uncharted microbes to drugs [23]. This approach involves juxtaposing microbial genomic sequences to spot drug-resistant genes and delving into drug mechanisms as well as microbial metabolic pathways [24]. However, these conventional strategies often grapple with intrinsic constraints. Laboratory research, for instance, can be hemmed in by costly equipment and an inability to authentically replicate *in vivo* conditions. Clinical observations and empirical treatments, while invaluable, depend on extensive case histories and prolonged observation, rendering swift summarization and diagnosis challenging. These challenges underscore the burgeoning interest in harnessing deep learning methodologies and technologies to explore microbial drug responses.

In recent years, the deployment of deep learning technologies within bioinformatics has surged [25–27], notably in studies examining the responses of microbes to drugs. Zhu *et al.* [28] constructed a microbe-drug response network by integrating microbial similarity, drug similarity and known microbe–drug networks, and then predicted the potential microbial responses to drugs hinged on KATZ measurements. Long *et al.* introduced a predictive framework rooted in graph convolutional neural networks (GCNs) to discern microbial responses to drugs. They enhanced this GCN by incorporating both conditional random fields and attention mechanisms, optimizing message propagation and aggregation processes [29]. Building on this, Long *et al.* formulated a comprehensive model grounded in graph networks and attention mechanisms. This model, drawing on microbe–drug networks from three distinct sources, merged node and graph-level attention mechanisms to compute potent node representations for microbes and drugs, subsequently predicting microbial responses to drugs [30]. Deng *et al.* harnessed variational graph autoencoders for the same purpose. By constructing a multimodal attribute graph—reflecting microbial genetic sequences, drug molecular structures and drug functional annotations, they trained their autoencoder, predicting microbial responses to drugs via a deep neural network [31]. Yang *et al.*[32] unveiled a multi-kernel fusion model anchored in GCN, extracting multi-layer embeddings to determine potential microbial responses to drugs. Drawing from graph contrastive learning, Tian *et al.* designed a structure-enhanced contrast learning model coupled with adaptive negative sampling. This model not only enhance node representation through contrastive learning but also selected the most pertinent negative samples for a multi-layer neural network classifier to investigate microbial responses to drugs [33]. These methods solely take into account the directly adjacent nodes, neglecting higher order neighboring nodes during the aggregation and updating processes. As a result, they fall short in extracting the robust representations of both microbes and drugs.

With the development of deep learning (especially GNN), there has been a surge in the adoption of related technologies. For message-passing techniques, methods based on subgraphs often have an edge over those based on entire graphs. This advantage stems from the fact that subgraph construction frequently incorporates high-order neighbors of nodes, subsequently enhancing topological information. Yet, it is important to highlight that subgraph-based approaches often demand significant memory and time resources. Fortunately, there are ways to reduce complexity while increasing performance. MinHash is a technique used to estimate the Jaccard similarity between sets [34]. It is widely used in the fields of big data and information retrieval, especially when directly calculating Jaccard similarity becomes very expensive. The core idea of MinHash is to use hash functions to sample set elements in a controllable manner. HyperLogLog is a probabilistic algorithm used to estimate the number of different elements in a set, also known as cardinality [35]. Compared with directly calculating cardinality, HLL uses much less memory than traditional methods, but at the cost of smaller accuracy losses.

Current research methodologies effectively derive precise representations of microbes and drugs from multi-source similarity or topological data, aiming to predict potential microbial responses to drugs. However, these strategies often overlook the influence of high-order neighborhoods on nodes, which can diminish prediction accuracy. Furthermore, these methods rely on the entire microbe-drug response graph for data propagation, leading to the challenge of ’over-smoothing’. In response, our approach marries deep autoencoders with subgraph enhancement techniques to introduce a novel model for predicting microbial responses to drugs. We extract robust node representations for microbes and drugs from their individual similarity matrices using deep autoencoders. Concurrently, we anchor each microbe (or drug) node within the established microbe-drug response network to derive their high-order subgraphs. Subsequently, the MinHash and HyperLoglog algorithms estimate the overlaps and cardinality between microbial and drug subgraphs. These metrics are then integrated with initial features, encoded by GCN, and a multilayer perceptron (MLP) classifier is employed to infer the potential responses of microbes to drugs. Empirical evaluations across diverse datasets demonstrate that our proposed JDASA-MRD model takes account more deeply into the topological neighborhood information of microbes and drugs, mitigates the ’over-smoothing’ issue and predicts microbial responses to drugs with heightened accuracy. Our contributions can be summarized as follows.

1. We merge deep autoencoders with subgraph enhancement techniques to propose the JDASA-MRD model, designed for efficiently predicting the potential responses of microbes to drugs.
2. Utilizing the MinHash and HyperLoglog algorithms, we estimate the overlap and cardinality count between microbial and drug subgraphs, deeply mine the multi-hop neighborhood topological information of nodes. In the GCN iteration, the node subgraph structure is integrated, while alleviating the ’over-smoothing’ issue.
3. We incorporate higher order neighbors beyond the microbe (or drug) subgraph, minimizing information loss at the node level and enhancing node representation.
4. We executed numerous comparative analyses and parameter tests on public datasets to demonstrate the effectiveness of the proposed JDASA-MRD model.

## MATERIALS AND METHOD

This section is divided into two main parts: initially, it outlines the data utilized in the study, followed by an in-depth discussion of the techniques underpinning the proposal of the JDASA-MRD model.

### Datasets

We commence by introducing the three published datasets of microbial responses to drugs: MDAD [36], aBiofilm [37] and DrugVirus [38] employed in our experiment. The MDAD dataset archives response records of 173 microbes to 1373 drugs, aBiofilm chronicles the responses of 140 microbes to 1720 drugs and DrugVirus documents the responses of 95 viruses to 175 drugs. In each dataset, the documented microbe-drug responses form a bipartite graph network. Its adjacency matrix, represented as $A$, has dimensions $N_m \times N_d$, where $N_m$ denotes the count of microbes and $N_d$ signifies the drug tally. If $A(i,j)$=1, it indicates the $i$th microbe reacts to the $j$th drug; otherwise, it signifies no observable stress response from the $i$th microbe to the $j$th drug. And we calculate the average number of 1-hop, 2-hop and 3-hop neighboring nodes for each node in the graph, and the statistics are shown in Table 1. Furthermore, the model gleans feature matrices from the similarity matrices of both microbes and drugs, details of which we will discuss subsequently.

**Table 1:** Detailed statistics of the datasets

datasets	MDAD	aBiofilm	DrugVirus
microbes	173	140	95
drugs	1373	1720	175
responses	2470	2884	933
1-hop	3.20	3.10	6.91
2-hop	262.54	416.31	64.44
3-hop	107.71	118.54	84.52

#### Microbe similarity matrix

Referring to prior studies [33], we construct a microbe similarity matrix that incorporates both microbe functional similarity and Gaussian interaction spectrum kernel-based similarity. These measures elucidate microbe similarity from dual perspectives. The computation process for microbe functional similarity is meticulously detailed and validated in the studies [29, 39]. As for the Gaussian interaction spectrum kernel similarity, it operates suppose that analogous microbes react with analogous drugs. Conveniently, consider matrix $A$ representing the microbe-drug response matrix. Here, $A(m_i)$ and $A(m_j)$ symbolize the interaction spectra for microbes $m_i$ and $m_j$, respectively. The similarity grounded in the Gaussian interaction spectrum kernel is computed as follows: 


(1) \begin{align*}& GM(m_i,m_j)=exp(-\eta_m||A(m_i)-A(m_j)||^2),\end{align*}

where $\eta _m$ is the normalized kernel bandwidth, calculated as follows: 


(2) \begin{align*}& \eta_m={\eta_m}^{\prime}/\left(\frac{1}{N_m}\sum_{i=1}^{N_m}||A(m_i)||\right).\end{align*}

After calculating the microbial Gaussian interaction spectrum kernel similarity matrix, it is jointly constructed with the microbial functional similarity matrix to form the final similarity matrix 


(3) \begin{align*}& S_m(m_i,m_j) = \begin{cases} \frac{FM(m_i,m_j)+GM(m_i,m_j)}{2} & if\ FM(m_i,m_j)\neq 0, \\ GM(m_i,m_j) & otherwise, \\ \end{cases}\end{align*}

#### Drug similarity matrix

Similarly, we construct a drug similarity matrix using both structural similarities and the Gaussian interaction spectrum kernel-based similarities. The structural similarity between drugs was calculated based on Hattori *et al.*’s methodology [40]. For two drugs $d_i$ and $d_j$, their similarity is denoted as $DS(d_i, d_j)$, which facilitates the construction of a structure-based drug similarity network. Adopting an analogous methodology as applied to microbes, we compute similarities using Gaussian interaction spectral kernels. Within the microbe-drug association matrix $A$, $A(d_i)$ and $A(d_j)$ characterize the interaction spectra for drugs $d_i$ and $d_j$, respectively. The Gaussian interaction spectrum kernel-based similarity is then determined as follows: 


(4) \begin{align*}& GD(d_i,d_j)=exp(-\eta_d||A(d_i)-A(d_j)||^2),\end{align*}

$\eta _d$ is the normalized kernel bandwidth 


(5) \begin{align*}& \eta_d={\eta_d}^{\prime}/\left(\frac{1}{N_d}\sum_{i=1}^{N_d}||A(d_i)||\right).\end{align*}

After calculating the drug Gaussian interaction spectrum kernel similarity matrix, it is jointly constructed with the drug functional similarity matrix to form the final similarity matrix 


(6) \begin{align*}& S_d(d_i,d_j)= \begin{cases} \frac{DS(d_i,d_j)+GD(d_i,d_j)}{2} & if \; DS(d_i,d_j)\neq 0, \\ GD(d_i,d_j) & otherwise. \\ \end{cases}\end{align*}

### Method

**Figure 1:**
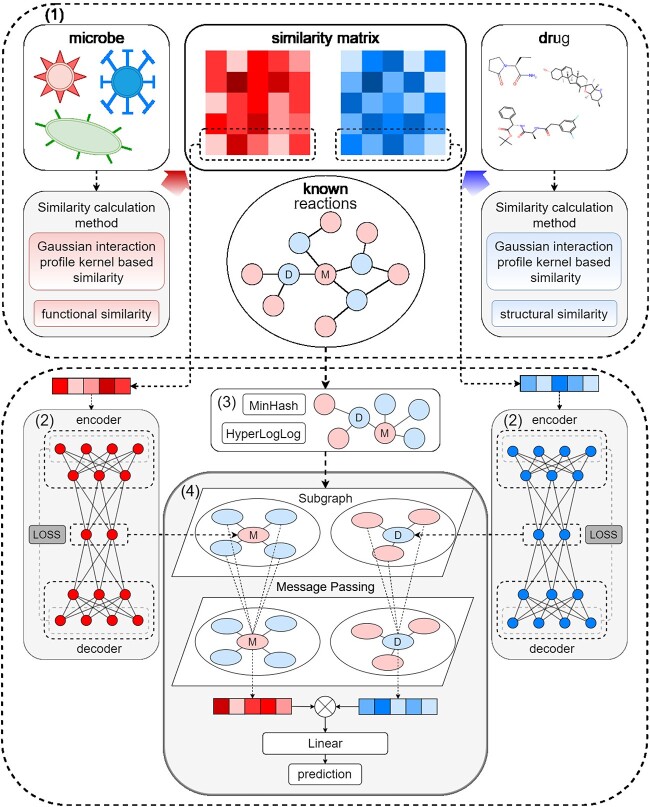
The architecture of the JDASA-MRD model is primarily divided into four distinct modules. In module (1), various similarity measure techniques are employed to formulate the similarity matrices for both microbes and drugs. In module (2), these similarity matrices are processed through two separate deep autoencoders to derive condensed representations for the microbes and drugs. For module (3), centering on each node in the microbe-drug response graph, a subgraph is extracted. Subsequently, the MinHash and HyperLogLog algorithms are utilized to approximate the node’s subgraph structural attributes. In module (4), the condensed microbe (or drug) features are merged with their subgraph-based structural attributes and then channeled into the message passing layer (like GCN) for aggregating and updating. Ultimately, features of the microbe–drug pair are computed, and potential responses of microbes to drugs are projected using MLPs.

In our research, we integrate deep autoencoders with subgraph structure augmentation to introduce the JDASA-MRD model, aimed at predicting microbes’ potential responses to drugs. As shown as Figure 1, the proposed JDASA-MRD model encompasses four primary components: (i) the Data Collection module, comprehensively detailed in Section 2.1; (ii) the Subgraph Structural Feature Extraction module; (iii) the Deep Autoencoder module and (iv) the GNN encoder module based on subgraph structure enhancement.

#### Deep autoencoder

Autoencoder is an unsupervised deep learning architecture, fundamentally comprising two components: the encoder and the decoder. It is frequently employed for tasks such as data dimensionality reduction and feature extraction. The autoencoder is adept at compressing the input data into a low-dimensional representation and then reconstructing the original data from this reduced form. Building on this concept, we utilize a deep autoencoder that incorporates multiple stacked encoder–decoder layers to project the similarity matrix of microbes (or drugs) into a low-dimensional space. For the drug-side autoencoder, the two encoding layer dimensions are set to 512 and 128, and the two decoding layer dimensions are set to 128 and 512, respectively. The activation function of each layer is set to RELU. For the microbe-side autoencoder, the two encoding layer dimensions are set to 128 and 64, and the two decoding layer dimensions are set to 64 and 128, respectively. The activation function of each layer is set to RELU. This approach not only effectively captures the accurate representations of microbes (or drugs) but also filters out extraneous noise. Within the encoder, a linear transformation is applied to map the input microbial (or drug) similarity feature matrix into a compressed space 


(7) \begin{align*}& H=g_{\theta 1} (X)=\psi (w_1X+b_1),\end{align*}

where 


(8) \begin{align*}& \psi(X) = \frac{1}{1+exp(-X)},\end{align*}

and $H$ denotes the condensed features derived from the encoding process, $w_1$ is the trainable weight matrix and $b_1$ is the bias matrix. In the decoder phase, the reduced-dimensional features are reconstructed using linear transformations 


(9) \begin{align*}& \widehat{X} = g_{\theta 2} (H) = \phi (w_2H+b_2),\end{align*}

where $\widehat{X}$ signifies the reconstructed node features, $w_2$ is the adjustable weight matrix and $b_2$ is the bias matrix. For a given node $i$, if its original features are denoted as $X_i$ and its reconstructed features as $\widehat{X}_i$, and the total node count is $N$, the mean squared error loss function measures the discrepancy between the input and reconstructed features as follows: 


(10) \begin{align*}& L(X, \widehat{X}) = \frac{1}{N}\sum_{i=1}^{N}(X_i - \widehat{X}_i)^2.\end{align*}

#### Subgraph structure extraction

In common GNN models, the aggregation and updating of nodes rely on their direct neighbors. This strategy partially ignores the influence exerted by a node’s higher order neighbors, thus compromising the quality of the node representation. Inspired by previous work [41], we consider fusing the high-order neighborhood structure information of nodes instead of increasing the number of layers of the GNN model. In the microbe-drug response graph $G = (M, D, E)$, $M$ represents the microbial set, $D$ represents the drug set and $E$ represents the response set of microbes to the drugs. When microbe $m$ exhibits stress in response to drug $d$, it is likely that the higher order neighborhood structures surrounding $m$ and $d$ have more overlaps. Guided by this suppose, our attention is primarily directed toward the higher order neighborhood structures of nodes. For simplicity, we encapsulate these higher order neighborhoods within a subgraph centered around the node.

Centering on each microbe (or drug) node, we extract its $h$-hop subgraph using the random walk algorithm. From this, we can quantify the overlap (similarity) between microbe $m$ and drug $d$ as follows: 


(11) \begin{align*} & S1_{m,d}[d_m,d_d]=\lvert N_{d_m,d_d}(m,d) \rvert \nonumber \\ &- \sum_{x \leq d_m,\; y \leq d_d, \; (x,y) \neq (d_m,d_d)} \lvert N_{x,y}(m,d) \rvert,\end{align*}

where 


(12) \begin{align*}& N_{d_m,d_d}(m,d) \equiv N_{d_m}(m) \cap N_{d_d}(d),\end{align*}

and the terms $d_m$ and $d_d$ denote the distances to microbe $m$ and drug $d$, both less than $h$. $N_{d_m}(m)$ and $N_{d_d}(d)$ correspond to the $h$-hop neighbor sets of microbe $m$ and drug $d$, respectively. $S1_{m,d}[d_m,d_d]$ signifies the aggregate count of nodes situated $d_m$ units from microbe $m$ and $d_d$ units from drug $d$. This information propagation strategy that relies on local subgraph structure mitigates the over-smoothing issue typically arising from multi-layered message propagation in GNNs.

Currently, our emphasis lies on the local neighborhood structure within the node-centered subgraph. Given the fixed hop count $h$, there is an inevitable loss of certain higher order neighborhood details. To compensate for this, we also account for neighborhood overlaps external to the subgraph 


(13) \begin{align*}& S2_{m,d}[k]=\lvert N_k(m)\rvert- S2_{m,d}[k-1]-\sum^k_{i=1} \sum^k_{j=1} S1_{m,d}[i,j],\end{align*}

where $S2_{m,d}[k]$ denotes the count of nodes that are $k$ units away from the microbe $m$ and more than $h$ units distant from the drug $d$.

In the given Equations 12 and 13, computing values for $\lvert N_{d_m,d_d}(m,d) \rvert $ and $\lvert N_k(m)\rvert $ is intricate. To simplify, we employ two approximation techniques: HyperLogLog and minHash. Let $a_m^k$ and $t_m^k$ represent the $h$-hop neighbors of microbe $m$ as estimated using HyperLogLog and minHash, respectively, with $a_m^k=max_{d \in N(m)}a_d^{k-1}$ and $t_m^k=min_{d \in N(m)}t_d^{k-1}$. With these approximations, $\lvert N_{d_m,d_d}(m,d) \rvert $ can be estimated as follows: 


(14) \begin{align*} \lvert N_{d_m,d_d}(m,d) \rvert & \equiv N_{d_m}(m) \cap N_{d_d}(d) \nonumber \\ & =J(N_{d_m}(m),N_{d_d}(d)) \cdot \lvert N_{d_m}(m) \cup N_{d_d}(d) \rvert \nonumber \\ & \approx H(t_m^{d_m},t_m^{d_m}) \cdot card(max(a_m^{d_m},a_d^{d_d})),\end{align*}

where $J$ denotes the Jaccard similarity between the two sets, while $H$ signifies the Hamming similarity, and $hyper$ stands for the HyperLogLog cardinality estimation function. In the given equation, MinHash is employed to approximate the Jaccard similarity between two sets, and HyperLogLog is leveraged to estimate the set cardinality. Moreover, $\lvert N_k(m)\rvert $ can be approximated as $hyper(a_m^k)$.

#### GNN encoder based on subgraph structure augmentation

In our research, the GNN encoder utilizes a standard message propagation mechanism. Yet, during the aggregation and update phase, information from the node’s subgraph structure is incorporated 


(15) \begin{align*} & e_{m,d}^l=\{S2_{m,d}[l], S1_{m,d}[d_m,l], S1_{m,d}[l,d_d]: \forall d_m,d_d < l\} \end{align*}

(16) \begin{align*} & x_m^l=\lambda^l(x_m^{l-1},aggregate_{d\in N(m)} \theta^l(x_m^{l-1},x_d^{l-1},e_{m,d}^l)), \end{align*}

where $\lambda $ and $\theta $ denote trainable linear functions, incorporating internal concatenate operations, while $aggregate$ signifies an aggregation function. $x^l_m$ and $x^l_d$ correspond to the node features of microbe $m$ and drug $d$ in the $l$th layer, with $x^0_m$ and $x^0_d$ being the initial features extracted by the deep autoencoder. $e_{m,d}$ embodies the edge features and serves to represent the weight of $(m,d)$. Based on Equations 16 and 17, message propagation transpires within the subgraph’s local structure. Compared with global message propagation, such localized propagation considerably mitigates the over-smoothing issue.

After $h$ propagation cycles, we obtain the final representations for the microbe node $m$ and the drug node $d$. By integrating the local structural feature of each node’s subgraph, we derive the representation for the microbe-drug response pair 


(17) \begin{align*}& p(m,d)=\psi (x_m^h \odot x_d^h, \{S2_{m,d}[k], S1_{m,d}[d_m,d_d]: \forall k, d_m, d_d \in [ h]\}),\end{align*}

where $\psi $ denotes trainable function, $\odot $ signifies Hadamard product and $[ h]=\{1,2,...,h \}$. After several propagation cycles, we obtain the final representations for the microbe node $m$ and the drug node $d$. By integrating the local structural feature of each node’s subgraph, we derive the representation for the microbe-drug response pair 


(18) \begin{align*}& \mathcal{L}=\sum_{m\in M,d\in D} (y(m,d)-1)\cdot log(1-p(m,d))-y(m,d)\cdot log(p(m,d)),\end{align*}

where $y(m,d)$ denotes the actual response of microbe $m$ to drug $d$. If a response exists, its value is 1; otherwise, it is 0.

## EXPERIMENT RESULTS

In this section, we delve into the performance evaluation of our proposed JDASA-MRD model through various aspects: First, we initiate by running the JDASA-MRD model through multiple iterations on three public datasets to ascertain its effectiveness. Second, by employing several cross-validation trials and adjusting the ratios for collecting negative samples, we juxtapose the efficacy of the JDASA-MRD model with other existing state-of-the-art models. Third, we undertake a series of parameter tests to affirm the model’s robustness and offer insights into optimal parameter configurations. Finally, we present a case study where we analyze the potential responses of microbes to two specific drugs: Curcumin and Epigallocatechin Gallate. To ensure a fair evaluation, the same standards, including positive and negative sample distributions and evaluation metrics ($AUC$, $AUPR$, $F1-Score$, $ACC$, $MCC$), are maintained throughout the experiments.

For comparison methods, we select the currently advanced DTIGAT [42], NIMCGCN [43], MMGCN [44], DTI-CNN [45] and SCSMDA [33] models.

1. DTIGAT [42] incorporates the self-attention mechanism into the microbe-drug response network. By leveraging GNN, it extracts node representations for both microbes and drugs to predict potential responses of microbes to drugs.
2. NIMCGCN [43] employs GCN technology to derive potential representations from the similarity matrices of both microbes and drugs. Subsequently, using a neural induction approach, it completes the microbe-drug response matrix and predicts potential responses.
3. MMGCN [44] utilizes GCN to compute the representations of microbes and drugs across various similarity perspectives. A multi-channel attention mechanism then adaptively adjusts these features to ascertain the potential response of microbes to drugs.
4. DTI-CNN [45] is a fusion of representation learning and GNN techniques. Initially, it calculates the correlation features of microbes and drugs from heterogeneous networks using the Jaccard coefficient. Then, dimensionality is reduced through an autoencoder, and potential response of microbes to drugs is predicted using GCN.
5. SCSMDA [33] constructs a network based on similarity and meta-paths. It harnesses a contrastive learning strategy to train representations of both microbes and drugs and employs a bespoke negative sampling strategy to enhance its predictive accuracy.

### Performance evaluation

**Figure 2:**
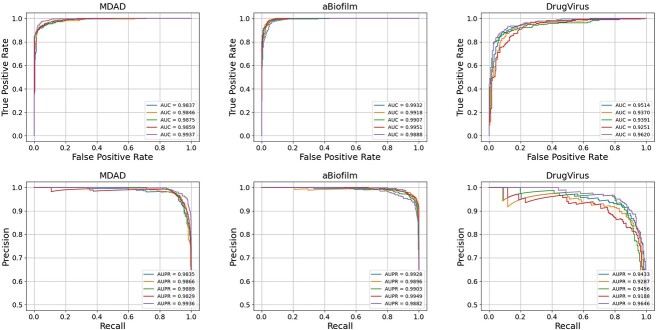
The models’ AUC and AUPR performance on the MDAD, aBiofilm and DrugVirus datasets through 5-fold cross-validation experiments.

To evaluate the performance of our proposed JDASA-MRD model, we conducted a 5-fold cross-validation on MDAD, aBiofilm and DrugVirus datasets, subsequently plotting the AUC against AUPR curves. As depicted in Figure 2, the JDASA-MRD model showcased commendable performance across the three datasets. Specifically, for the MDAD, aBiofilm and DrugVirus datasets, the model achieved average AUC values of 98.71%, 99.19% and 94.29%, respectively. Similarly, the average AUPR values were impressive, registering at 98.71%, 99.12% and 94.02% for each dataset, respectively. These results validate that the JDASA-MRD model is adept at accurately predicting the responses of microbes to drugs across diverse datasets.

**Figure 3:**
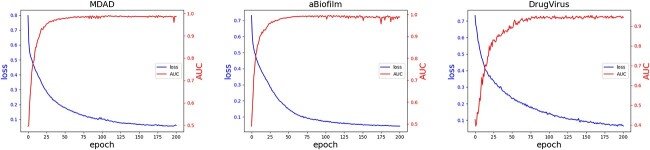
The curves of training loss and AUC performance versus number of training epochs.

Moreover, we undertook 200 training iterations for the model, illustrating both the loss value for each iteration and the AUC value of the test set in Figure 3. The trends depicted in the figure reveal that the model began exhibiting predictive capabilities after merely 25 training iterations. By the 50th iteration, its predictive prowess had peaked. As training continued beyond this point, the loss value exhibited a consistent decline, while the model’s AUC performance largely plateaued. Notably, there is no discernible overfitting, attesting to the robustness and stability of the JDASA-MRD model. This stability is likely attributable to the model’s use of local subgraph structures for message propagation, effectively mitigating over-smoothing issues.

### Comparison with other method

**Figure 4:**
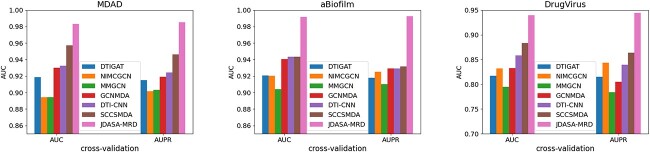
Comparison of AUC and AUPR performance of multiple models on MDAD, aBiofilm and DrugVirus datasets. The proposed JDASA-MRD model significantly outperforms the others.

Constrained by various real-world factors, only a minuscule portion of microbial responses to drugs has been confirmed experimentally, leaving the majority still uncharted. As a result, false negative samples can significantly hamper model performance. To evaluate how various models cope with these false negatives, we adjusted the ratios of positive to negative samples. For the experiment, we chose two specific ratios, 1:1 and 1:5, and employed AUC and AUPR as the evaluation metrics. The outcomes of these tests are presented in Table 2 and Figure 4.

**Table 2:** Performance comparison of various models across three datasets with negative sampling ratios of 1:1 and 1:5

		1:1		1:5	
Datasets	Models	AUC	AUPR	AUC	AUPR
	DTIGAT	91.85%	91.49%	91.85%	91.49%
	NIMCGCN	89.44%	90.16%	89.44%	90.16%
MDAD	MMGCN	89.43%	90.33%	89.43%	90.33%
	GCNMDA	92.99%	91.92%	92.99%	91.92%
	DTI-CNN	93.25%	92.42%	93.25%	92.42%
	SCSMDA	95.73%	94.64%	95.73%	94.64%
	JDASA-MRD	98.78%	98.71%	98.31%	98.54%
	DTIGAT	92.05%	91.79%	91.56%	75.65%
	NIMCGCN	92.01%	92.51%	91.43%	76.26%
aBiofilm	MMGCN	90.42%	91.03%	90.72%	75.84%
	GCNMDA	94.07%	92.91%	93.74%	76.23%
	DTI-CNN	94.36%	92.91%	94.12%	78.91%
	SCSMDA	94.36%	93.16%	95.59%	79.71%
	JDASA-MRD	99.19%	99.12%	96.45%	93.16%
	DTIGAT	81.69%	81.52%	80.01%	46.36%
	NIMCGCN	83.19%	84.38%	84.24%	52.80%
DrugVirus	MMGCN	79.46%	78.40%	77.91%	47.64%
	GCNMDA	83.30%	80.47%	83.66%	47.88%
	DTI-CNN	85.81%	83.96%	84.66%	56.44%
	SCSMDA	88.34%	86.37%	87.57%	57.77%
	JDASA-MRD	94.29%	94.02%	89.93%	71.83%

The data in Table 2 reveal that all models performed optimally when the ratio of positive to negative samples was 1:1. With a ratio of 1:5, the majority of models saw their performance significantly diminished due to the influence of false negatives. Notably, our proposed JDASA-MRD model delivered the best outcomes at both 1:1 and 1:5 ratios. Figure 4 graphically underscores the superior performance of the JDASA-MRD model over its counterparts. Additionally, the JDASA-MRD model demonstrates minimal performance degradation at the 1:5 ratio, suggesting its resilience against the impact of false negatives.

In direct comparison with the runner-up model, SCSMDA, the JDASA-MRD model showcased a pronounced enhancement in performance at both 1:1 and 1:5 sample ratios. Focusing on the AUPR metric across the three distinct datasets: MDAD, aBiofilm and DrugVirus, the JDASA-MRD model outpaced the SCSMDA model by 4.07%, 5.96% and 7.65%, respectively, at a 1:1 ratio. This lead further widened at a 1:5 ratio, with the JDASA-MRD model outperforming by 3.9%, 13.45% and 14.06%. Such results emphasize that, even under sample imbalances, the JDASA-MRD model maintains commendable performance. The findings also attest to the model’s ability to achieve elevated precision and recall rates, its resilience against the adverse effects of false negatives and its accurate inference of potential microbial responses to drugs.

**Figure 5:**
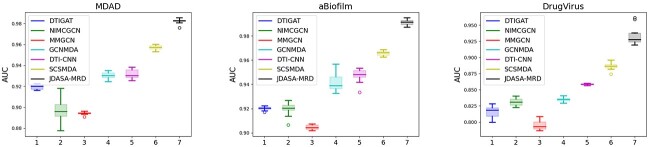
Results of 10 5-fold cross-validation experiments of the models on MDAD, aBiofilm and DrugVirus datasets. The proposed JDASA-MRD model consistently demonstrates a significantly higher average AUC value than other models, with minimal variance.

To evaluate the model’s stability, we executed 10 cross-validation trials for each model, with the outcomes presented in Figure 5. The box plot results reveal that the average AUC value of the JDASA-MRD model across the three datasets significantly surpasses that of the suboptimal model. Concurrently, the variation in its AUC values for these datasets remains notably low. These findings suggest that the JDASA-MRD model is not only accurate but also consistently reliable in predicting potential microbial responses to drugs.

Furthermore, the SCSMDA model employs structural enhancement and contrastive learning strategies, delivered second-best performance across all three datasets. However, its reliance on a conventional GNN encoder may predispose it to over-smoothing. This model also takes the direct approach of using the similarity matrices of microbes and drugs as the nodes’ initial representations. In contrast, the JDASA-MRD model we focus on information propagation based on the local subgraph structure, employing a deep autoencoder to derive the initial node representations for both microbes and drugs.

### Ablation experiment

#### Impact of different GNN layer

In our model, the GNN layers used for message passing can be diverse. We evaluated four typical GNN models, such as GCN, GIN, GAT and SAGE, on three datasets, as shown in Table 3. In the MDAD and aBiofilm datasets, the performance differences of each GNN model are small. This may be because the internal distribution of the two datasets is relatively even and thus is less affected by different GNN encoders. On the contrary, in the DrugVirus dataset, the performance of each GNN model differs slightly. This may be because the internal data volume of this dataset is small and it is more susceptible to the impact of error data, making it very sensitive to different GNN encoders. Generally speaking, using different GNN encoding layers will not have an extremely obvious impact on the model, such as a performance difference of more than 5%. At the same time, no matter which GNN encoding layer is adopted, the performance of the proposed model will be better than the sub-optimal model. Therefore, GNN encoding layers can be conveniently selected for new datasets.

**Table 3:** Results of different GNN layer on JDASA-MRD on MDAD, aBiofilm and DrugVirus datasets

Datasets	GNN layer	AUC	AUPR	ACC	SEN	PRE	SPE	MCC	F1
	GCN	98.79%	98.91%	94.03%	90.28%	97.59%	97.77%	88.30%	93.80%
MDAD	GIN	98.59%	98.71%	92.71%	87.65%	97.52%	97.77%	85.87%	92.32%
	GAT	98.42%	98.60%	91.90%	85.83%	97.70%	97.98%	84.43%	91.38%
	SAGE	98.29%	97.99%	92.91%	88.26%	97.32%	97.57%	86.20%	92.57%
	GCN	99.21%	99.15%	92.88%	87.50%	98.05%	98.26%	86.27%	92.48%
aBiofilm	GIN	98.88%	98.77%	93.14%	88.54%	97.51%	97.74%	86.65%	92.81%
	GAT	98.72%	98.84%	91.93%	85.42%	98.20%	98.44%	84.57%	91.36%
	SAGE	98.65%	98.05%	93.06%	89.41%	96.44%	96.70%	86.34%	92.79%
	GCN	93.90%	94.68%	87.37%	77.42%	96.64%	97.31%	76.26%	85.97%
DrugVirus	GIN	95.35%	96.09%	88.44%	80.65%	95.54%	96.24%	77.83%	87.46%
	GAT	93.39%	92.93%	84.95%	74.73%	93.92%	95.16%	71.40%	83.23%
	SAGE	91.09%	90.95%	82.80%	73.12%	90.67%	92.47%	66.86%	80.95%

#### Impact of subgraph structural feature

To explore the impact of subgraph structural feature on model performance, we designed several sets of ablation experiments, and the results are shown in Table 4. ’w/o SSF’ means that the model does not adopt the subgraph structural features of the node but only uses the features of the node itself. For the MDAD and aBiofilm datasets, the results show that all indicators drop slightly after removing subgraph structural features. For the DrugVirus dataset, after removing the subgraph structural features, the performance dropped significantly. These results prove that subgraph structural features can indeed enhance the performance of the model.

**Table 4:** The impact of subgraph structural feature on model performance

Datasets	set	AUC	AUPR	ACC	SEN	PRE	SPE	MCC	F1
	w/o SSF	98.47%	98.71%	93.22%	89.88%	96.31%	96.56%	86.63%	92.98%
MDAD	JDASA-MRD	98.79%	98.91%	94.03%	90.28%	97.59%	97.77%	88.30%	93.80%
	w/o SSF	98.73%	98.59%	91.15%	84.03%	97.98%	98.26%	83.14%	90.47%
aBiofilm	JDASA-MRD	99.21%	99.15%	92.88%	87.50%	98.05%	98.26%	86.27%	92.48%
	w/o SSF	91.51%	92.53%	84.95%	75.27%	93.33%	94.62%	71.24%	83.33%
DrugVirus	JDASA-MRD	93.90%	94.68%	87.37%	77.42%	96.64%	97.31%	76.26%	85.97%

### Parameter experiment

To evaluate the robustness of the JDASA-MRD model, we carried out several parameter experiments on three datasets: MDAD, aBiofilm and DrugVirus. Initially, while maintaining consistency in other experimental conditions, we gauged how the number of training iterations for the autoencoder influenced the JDASA-MRD model’s performance. Subsequently, we delved into the interplay between the initial input feature dimension of the node and the feature dimension of the node’s subgraph structure. In our concluding exploration, we centered on the ramifications of extracting structural features from subgraphs of varied orders on the model’s efficacy.

#### Impact of autoencoder training epochs

**Figure 6:**
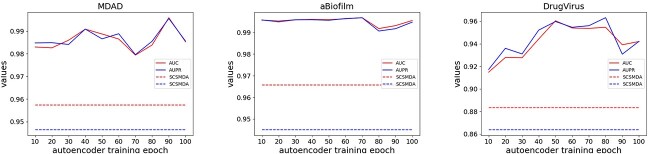
The impact of deep autoencoder training epochs on model performance.

Within the JDASA-MRD model, an autoencoder initiates by encoding the similarity matrix of microbes (and drugs), subsequently extracting the node representations of these microbes (and drugs). These representations then serve as initial features in the ensuing message propagation phase. This study primarily delves into the influence of the autoencoder’s training epochs on the quality of the initial node representations and, in turn, the overarching performance of the model. We carried out 10 experiments across three datasets, using the SCSMDA model as a reference benchmark, as depicted in Figure 6.

In summary, the JDASA-MRD model demonstrates consistently stable performance across the three datasets. On the MDAD and DrugVirus datasets, there is a notable enhancement in the model’s efficacy with the escalation in the training epochs. The pinnacle of performance for the JDASA-MRD model is achieved at 90 and 50 training epochs for the MDAD and DrugVirus datasets, respectively, after which there is a decline. This observation suggests that increasing the autoencoder’s training epochs to an optimal count can significantly enhance the model’s outcome. For maintained model robustness, it is pragmatic to set the training epochs around the ballpark of 100. Notably, across all three datasets, the JDASA-MRD model outperforms the baseline substantially.

#### Impact of feature dimension

During the JDASA-MRD model’s message propagation phase, the autoencoder-derived initial features of microbes (and drugs) are concatenated with the subgraph structural features of these microbes (and drugs) to form the primary input features. The dimensions of these features potentially sway the fidelity of the microbes (and drugs) representations. Consequently, experiments are designed to probe the combined effects of the autoencoder’s output dimension and the subgraph structure’s feature dimension on the predictive performance of the model. This is aimed at furnishing insights for optimal parameter configuration.

**Figure 7:**
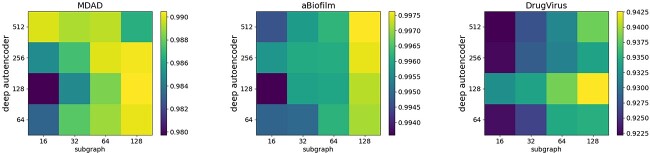
The impact of feature dimensions on model performance. The horizontal axis denotes the dimensionality of the extracted subgraph structural features, while the vertical axis indicates the feature dimension output from the deep autoencoder.

From the data illustrated in Figure 7, it becomes evident that varying feature dimensions influence the model’s efficacy. On the MDAD and DrugVirus datasets, the zenith of the model’s performance is realized with a feature dimension set at 128. For the aBiofilm dataset, optimal outcomes materialize when the subgraph structure’s feature dimension stands at 128, coupled with an autoencoder output dimension of 512. In general, increasing feature dimensions carefully can elevate the precision of microbes (and drugs) representations. When confronted with unfamiliar data, it is prudent to anchor the feature dimensions within the realms of 128, 256 or 512.

#### Impact of subgraph size

**Figure 8:**
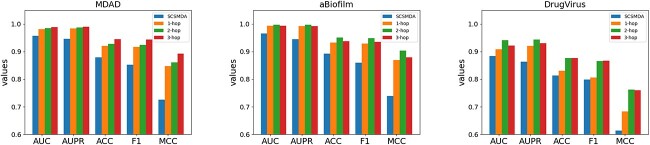
The impact of subgraph size on model performance.

The JDASA-MRD model operates its message propagation leveraging the subgraph structure of nodes. Understandably, the subgraph structure can impart considerable influence on the model’s performance. In our experiments, we extract the 1-hop, 2-hop and 3-hop subgraphs centered on nodes across three datasets, with the SCSMDA model serving as the reference baseline. The outcomes are delineated in Figure 8. For the MDAD dataset, the model exhibits heightened performance with the extraction of 3-hop subgraphs. However, for the aBiofilm and DrugVirus datasets, the pinnacle of performance is achieved when 2-hop subgraphs are utilized.

This observation underscores a couple of salient points: Extracting overly small subgraphs might lead to an omission of information, while overly large subgraphs could harm the performance with redundant data. Therefore, to accommodate unfamiliar datasets, it is astute to default to the extraction of 2-hop subgraphs. Notably, irrespective of the chosen parameter configurations, the JDASA-MRD model consistently outshines the performance of the second-best SCSMDA model.

#### Case study

We selected curcumin and epigallocatechin gallate for in-depth case studies to evaluate the performance of the JDASA-MRD model under isolation conditions. Curcumin is a turmeric compound with strong antioxidant and anti-inflammatory properties, which may help treat neurodegenerative diseases, reduce the risk of cardiovascular disease and fight cancer. It has also been studied for antidepressants and anti-aging and can be consumed as a supplement or food, although its bioavailability is low, consuming Curcumin is usually safe. Epigallocatechin gallate is the main active ingredient of green tea, with antioxidant, anti-inflammatory and anticancer properties, which may help reduce the risk of cardiovascular disease, weight loss and neuroprotection. However, its bioavailability is limited, and high intake may have side effects.

During the model training, we exclude microbes related to these two drugs. In the testing phase, we try to predict the microbes responsive to Curcumin and Epigallocatechin Gallate. We rank these based on predicted probabilities and spotlight the top 20 microbes, as outlined in Tables 5 and 6. The results garnered from the table suggest that a majority of the forecasted microbes exhibit stress responses to both Curcumin and Epigallocatechin Gallate, corroborated by the MDAD database. Additionally, microbes Acinetobacter nosocomialis and Aeromonas hydrophila unverified in the MDAD database do show evidence of stress response to Curcumin as referenced in studies [46, 47]. And in Table 6, Microbe Yersinia enterocolitica, which is not verified in the MDAD database, is shown to have a stress response to Drug Epigallocatechin Gallate in previous study [48]. These observations reflect the superior performance of the JDASA-MRD model in isolation scenarios, which can explore the potential response of microbes to drugs and then guide drug treatment plans.

**Table 5:** The top 20 microbes predicted by the proposed JDASA-MRD model to be stress-responsive to the drug Curcumin

microbes	MDAD	microbes	MDAD
Pseudomonas aeruginosa	definite	Aeromonas hydrophila	definite
Burkholderia cenocepacia	definite	Citrobacter freundii	definite
Burkholderia multivorans	definite	Enterobacter cancerogenus	definite
Escherichia coli	definite	Enterobacter ludwigi	definite
Vibrio harveyi	definite	Klebsiella variicola	definite
Vibrio parahaemolyticus	definite	Pseudomonas japonica	definite
Vibrio vulnificus	definite	Raoultella ornithinolytica	definite
Streptococcus mutans	definite	Candida albicans	definite
Proteus mirabilis	definite	Acinetobacter nosocomialis	undefinite
Serratia marcescens	definite	Aeromonas hydrophila	undefinite

‘define’ indicates that it has been validated in the database.

**Table 6:** The top 20 microbes predicted by the proposed JDASA-MRD model to be stress-responsive to the drug Epigallocatechin Gallate

microbes	MDAD	microbes	MDAD
Staphylococcus aureus	definite	Enterobacter ludwigi	definite
Staphylococcus epidermidis	definite	Klebsiella variicola	definite
Burkholderia cenocepacia	definite	Pseudomonas japonica	definite
Burkholderia multivorans	definite	Raoultella ornithinolytica	definite
Candida albicans	definite	Serratia marcescens	definite
Eikenella corrodens	definite	Streptococcus pneumoniae	definite
Streptococcus mutans	definite	Thermus thermophilus	definite
Aeromonas hydrophila	definite	Vibrio anguillarum	undefinite
Citrobacter freundii	definite	Vibrio harveyi	undefinite
Enterobacter cancerogenus	definite	Yersinia enterocolitica	undefinite

‘define’ indicates that it has been validated in the database.

We studied related drugs that inhibit the microbes *Escherichia coli* and Pseudomonas aeruginosa. *Escherichia coli* is a type of bacteria commonly found in the intestines. These bacteria are mostly harmless and are part of the normal gut microbiota of humans and other warm-blooded animals. But there are also some variants of *E. coli* that can cause food poisoning, urinary tract infections or other illnesses. *Pseudomonas aeruginosa* is a Gram-negative rod-shaped bacterium. It is a species of the genus *Pseudomonas* and is widely distributed in natural environments such as soil, water and plant surfaces. It has strong pathogenicity, especially for individuals with low immune function. The discovery of potential pharmaceutical inhibitors can lead to the development of new treatment options.

The results in Tables 7 and 8 show that the proposed model accurately predicts the drugs that inhibit these two microbes, respectively, and most of the drugs are verified in the database. In Table 7, although Sulfamoxole has not been verified in the database, relevant work [49] has demonstrated its inhibitory activity against *E. coli*. Similarly, in Table 8, there is also studies [50, 51] proving that Biphenyl and Phenytoin drugs have antibacterial activity against *P. aeruginosa*. In summary, the proposed model can indeed discover the potential response of microbes to drugs, thereby promoting new treatment options.

**Table 7:** Top 20 drugs predicted to have antibacterial activity against *E. coli* using the proposed JDASA-MRD model

drug	MDAD	drug	MDAD
Benzylpenicillin	definite	Nitrofural	definite
Bergamottin	definite	Nobiletin	definite
beta-sitosterol glucoside	definite	Oxytetracycline	definite
Betulinic acid	definite	p-Coumaric acid	definite
Caffeic Acid	definite	Sulfamoxole	undefinite
Carabrone	undefinite	Sulfanilamide	definite
Carboxymethyl chitosan	definite	Sulfaphenazole	definite
Myristic acid	definite	Bac8C	undefinite
Neomycin	definite	Carabrol	undefinite
Netilmicin	definite	Josamycin	undefinite

‘define’ indicates that it has been validated in the database.

**Table 8:** Top 20 drugs predicted to have antibacterial activity against Pseudomonas aeruginosa using the proposed JDASA-MRD model

drug	MDAD	drug	MDAD
Betulin	definite	TZD-C8	definite
Biphenyl	undefinite	Zinc oxide	definite
Baicalein	definite	ZnO nanoparticles	definite
BMAP-28	definite	Vitexin	definite
Chlorpromazine	definite	Picolinic acid	definite
Cobalt Ion	definite	Pinguisenol	definite
Seg6D	definite	Pleurocidin	definite
Silver nanowires	definite	Phenytoin	undefinite
Sodium ascorbate	definite	Myxinidin3	definite
Tetrazol-1-yl	definite	Myristoyl-DL-carnitine	definite

‘define’ indicates that it has been validated in the database.

## CONCLUSION

Microbes often exhibit stress responses when exposed to certain drugs. Delving into these potential responses can drive advancements in drug discovery, antibiotic resistance, vaccine research and other related fields. We have reviewed both experimental and computational methodologies in this context. While experimental methods provide invaluable insights, they grapple with challenges such as high costs and time consumption. On the other hand, computational techniques that utilize deep learning and graph neural networks are adept at deriving precise node representations from sparse topologies, facilitating the discovery of potential microbial responses to drugs. Yet, these techniques face three primary limitations. First, they commonly employ randomized initial node representations, compromising data representational accuracy. Second, the impacts of high-order node neighborhoods are often overlooked, resulting in an incomplete extraction of node information. Third, by heavily relying on the entire graph for information propagation, they are susceptible to over-smoothing.

There, we plan to alleviate these problems in the following aspects. First, we harness the capabilities of deep autoencoder technology to craft robust initial node representations for both microbes and drugs. Second, the MinHash and HyperLoglog algorithms are employed to account the overlap and cardinality between microbe and drug subgraphs, enabling a deep exploration of the node’s multi-hop neighborhood topological information. Concurrently, we derive higher order neighborhood information beyond subgraphs to enhance node topological information. Third, by propagating messages grounded in initial node representations and localized subgraph structure, we alleviate the over-smoothing issue. To this end, our research marries deep autoencoder technology with subgraph structural feature extraction to propose the JDASA-MRD model, aimed at predicting potential microbial responses to drugs. Our comprehensive experiments and case studies on public datasets stand as testament to the model’s performance. Relevant case analysis proves that the JDASA-MRD model can effectively infer the unknown response of microbes to drugs, in order to help guide drug treatment plans.

**Key Points**

- We combine deep autoencoders with subgraph techniques to design the JDASA-MRD model for Inferring microbial responses to drugs.
- Using MinHash and HyperLoglog algorithms, we estimate the overlap and cardinality between microbe and drug subgraphs and extract multi-hop topological information.
- Information propagation in GCN relies on the node subgraph structure, which alleviates the ’over-smoothing’ problem.
- By including higher order neighbors outside the subgraph, we reduce node information loss and enhance node representation.

## Data Availability

Our data and code are publicly available at: https://github.com/ZZCrazy00/JDASA-MRD.

## Author Biographies

**Zhecheng Zhou** studies at College of Data Science and Artificial Intelligence, Wenzhou University of Technology.

**Linlin Zhuo** works at College of Data Science and Artificial Intelligence, Wenzhou University of Technology.

**Xiangzheng Fu** works at College of Computer Science and Electronic Engineering, Hunan University.

**Quan Zou** works at Institute of Fundamental and Frontier Sciences, University of Electronic Science and Technology of China.
